# Social Impact of Psychological Research on Well-Being Shared in Social Media

**DOI:** 10.3389/fpsyg.2020.00135

**Published:** 2020-02-26

**Authors:** Cristina M. Pulido, Liviu-Catalin Mara, Vladia Ionescu, Teresa Sordé-Martí

**Affiliations:** ^1^Department of Journalism and Communication Sciences, Autonomous University of Barcelona, Barcelona, Spain; ^2^Department of Business Management, University of Rovira and Virgili, Tarragona, Spain; ^3^Department of Sociology, University of Barcelona, Barcelona, Spain; ^4^Department of Sociology, Autonomous University of Barcelona, Barcelona, Spain

**Keywords:** social impact, social media, well-being, psychological research, SISM methodology

## Abstract

The purpose of this article is to demonstrate how the Social Impact in Social Media (SISM, hereinafter) methodology applied in psychological research provides evidence for the visibility of the social impact of the research. This article helps researchers become aware of whether and how their improvements are capturing the interest of citizens and how citizens are applying such evidence and obtaining better outcomes, in this case, in relation to well-being. In addition, citizens can access the latest evidence on social media and act as channels of communication between science and social or personal networks and, in doing so, they can improve the living conditions of others. This methodology is also useful for agencies that support researchers in psychology with financial assistance, which can use it to evaluate the social impact of the funds that they invest in research. In this article, the 10 studies on well-being were selected for analysis using the following criteria: their research results led to demonstrable improvement in well-being, and these improvements are presented on social media. We applied the social impact coverage ratio to identify the percentage of the social impact shared in social media in relation to the total amount of social media data collected. Finally, examples of quantitative and qualitative evidence of the social impact of the research on well-being are presented.

## Introduction

One of the current trends in research evaluation is to measure the social benefits of the research results by considering the social impact achieved through the implementation of evidence that guarantees improvements in different areas, which increase the quality of people’s lives (Reale et al., 2018). Psychological research is one of the disciplines that can contribute to different societal goals. It is one of the research areas that must be taken into consideration because the potential impact can improve people’s living conditions. This study shows how this discipline contributes to social impact through the analysis of one of its specific fields, well-being research, measured through the SISM methodology.

The roots of well-being research are anchored in ancient Greece, where philosophers focused on how to achieve “the good life,” which we currently call well-being (Stoll, 2014). Since then, the search for happiness or the meaning of life has been a constant topic handled by different disciplines; today, we have diverse scientific evidence demonstrating that “well-being” has a direct impact on people’s health (Hajek and Helmut König, 2019; Van de Cauter et al., 2019). Some authors even include happiness as a closely adjacent factor to well-being, and we have added this concept when papers refer directly to it or when the social media data analyzed include it too. This paper aims at corroborating how the application of the SISM methodology can identify evidence of the social impact of well-being research. SISM collects and analyses social media data that mentions the evidence of research that improves people’s lives and health and is capturing the interest of citizens.

Thus, this paper first reviews some key contributions of well-being research from the psychological perspective that are in line with one of the Sustainable Development Goals – SDG3: ensure healthy lives and promote well-being for all at all ages (World Health Organization [WHO], 2015; UNDP, 2017) and examines how social media plays a crucial role in capturing the interest of citizens with regard to the improvements that science is delivering to society. The second part presents the results extracted through the application of the SISM methodology to identify the sharing in social media of social impact evidence that has captured citizens’ interest. We selected 10 competitive projects on well-being research or related aspects that are present in social media. Next, we analyzed all the social media data shared to identify evidence of real or potential social impact. The results show that the research projects analyzed shared social impact evidence on social media and some of them achieved a high level of citizen interest, among other results. Future research lines are proposed in the final section of this article.

### Psychological Research on Health and Well-Being in Line With SDG3

Researchers from around the world need to pay attention to how their research projects contribute to societal goals. One of the ways to ensure that your research is answering these challenges is to review the priorities of the funding institutions, such as the European Commission in the case of Europe, or to review the priority goals of other international institutions, such as the United Nations (UN). In this last case, the list of Sustainable Development Goals (UNDP, 2017) represents a common agenda for researchers from different disciplines and countries. In this sense, there is a prominent field of psychological research focused on well-being that addresses a specific aim of SDG3: ensure healthy lives and promote well-being for all at all ages. This global goal contains one specific target (3.4): “(.) promote mental health and well-being” (World Health Organization [WHO], 2015). Considering this goal, the focus of this study is on well-being and not on subjective well-being. The literature differentiates between objective well-being, which depends on goods and values that go beyond the individual’s endorsement, and subjective well-being, where individuals decide what is good for their own lives (Bloodworth and McNamee, 2007). Consequently, subjective well-being includes (Diener et al., 1999), “people’s emotional responses, domain satisfactions, and global judgments of life satisfaction” (277). More broadly, well-being is a multidimensional phenomenon that integrates biological, psychological, social, and spiritual dimensions (Moreira et al., 2014).

Research on well-being has focused on identifying the aspects that contribute to individual life satisfaction and the relation established with the social environment. Therefore, research from other disciplines, such as sociology, can also contribute to this area, for instance, by looking at how trust in institutions (Barbalet, 2019) affects individuals’ well-being. A step forward was the discovery that those elements that guarantee well-being are understood as life expectation and satisfaction, but another advancement in recent years has been the demonstration that well-being directly influences individuals’ health. Well-being is not merely an aspect of happiness or a meaningful life; whether or not people find happiness, their well-being directly affects their mental and physical health (Jans-Beken et al., 2019). Moreover, well-being also has consequences for people’s quality of life and even their life expectancy (Evans and Soliman, 2019). Additionally, the impact of well-being affects other psychological factors, such as self-esteem and self-efficacy, that have a direct impact on self-confidence (Jaaffar et al., 2019).

In this regard, well-being research is crucial for improving individuals’ lives, and the identification of social impact evidence is necessary to understand what contributes to improving people’s lives and what does not. In fact, there is a constant denunciation of the existence of pseudoscience books about well-being that could promote negative impacts on people’s health. One example of this fact was described in a piece for the American Psychological Association’s Good Company blog, where authors reported that internet searches for the word “Happiness” on Amazon produced more than 92,000 hits, and this number is constantly increasing. Furthermore, a quick look at the best-sellers among these books shows that they are not evidence-based (Grawicht and Ballard, 2019). Therefore, the need to identify and disseminate evidence of the social impact of well-being research is not only a scientific need for the advancement of knowledge. It is also necessary to provide evidence to people that truly contributes to improving their well-being and even their physical and mental health and reducing risks to their health or well-being. Thus, health psychologists are bringing to light contributions to well-being research, examining the biological, social, and psychological factors that influence health and illness. These researchers build knowledge about how to achieve well-being according to the standards of the American Psychological Association. In this line, the World Health Organization, which defined health as “a state of complete physical, mental, and social well-being, not merely the absence of the illness,” developed two instruments for measuring quality of life (the WHOQOL-100 and the WHOQOL BREF) by considering individuals’ perceptions of their position in life and by taking into account the social context in which they live. These instruments have been used by different researchers for measuring the well-being aspects of health impact. This is an example of health and psychology researchers working together to advance this area of research.

For instance, more than 300 million people of all ages suffer from depression according to the data delivered by the World Health of Organization (World Health Organization [WHO], 2018). Some of the data collected include information about how well-being has a direct impact on the reduction of the risks faced when suffering from depression, which is a common mental disorder. One research finding highlights that engagement in productive activities, such as volunteering, in persons with a physical disability increases well-being, which directly impacts risks related to being depressed (Fekete et al., 2019). At the same time, this result indicates that it is important to “promote targeted interventions considering all the factors personal and psychological resources, reducing environmental barriers, and creating access to outside home activities” (Fekete et al., 2019, p. 7). Other results provide evidence of the direct impact of being engaged in meaningful activities on psychological well-being (Hooker et al., 2019). In this sense, it is relevant to identify the evidence that guarantees well-being to give people the opportunity to apply that particular evidence to promote better physical and mental health.

### Well-Being Research Through the Collection of Social Media Data

According to recent data, 3.499 billion people, of the approximately 7.697 billion people in the world, are active social media users (Hootsuite, 2019). Because of its massive spread, social media presents new issues but also opportunities for research in different areas and with different actors using technology. Thus, the internet offers the possibility for user-generated content that helps to organize and simplify the amount of available information (Kapoor et al., 2018) and thus increases the potential impact of a specific piece of information.

The internet presents many advantages for psychological research due to its continuous expansion and technological advancement. Some of these advances help scientists reach more diverse and larger samples, reducing the costs and time of fieldwork and the enabling development of novel research tools (Gosling and Mason, 2015). These two authors made an extensive study of the main types of research done using the internet, and they came up with three categories: translational, phenomenological, and novel. The translational studies are those that implement traditional methods in psychology through the internet, and the phenomenological study phenomena generated or disseminated by the internet. In contrast, innovative studies are those that allow the study of psychological topics from new perspectives offered by the internet (Gosling and Mason, 2015).

The amount of critical data that researchers can obtain from social media is huge. The use of data collection and analysis techniques that can extract useful results for different scientific purposes from social media has increased lately, with many new studies published in recent years. The use of social media as a dataset for different research purposes has increased in the last decade (Kapoor et al., 2018). Using a systematic review of the literature, Kapoor et al. (2018) highlight that among the most common topics investigated by the academic community are emotional, social, and health concerns, which are all connected with the focus on well-being research. Finally, most of the studies in their sample used social exchange, network, and organization theories, and they deal with the behavioral side of social media, reviews, and the incorporation of social media for marketing and organizational purposes. The bulk of the most recent psychological research on well-being in social media has been quantitative and used one or more scales via online questionnaires, which means that they are mostly transitional studies, in terms of Gosling and Mason (2015). Nonetheless, these studies present specific features of the other two types of studies, like considering the role of social media in generating and disseminating certain phenomena (e.g., addiction) or offering new perspectives, like an eye-tracking methodology (Hussain et al., 2019).

Atroszko et al. (2018) used one scale to study the relationship between Facebook addiction and psychosocial functioning and different dimensions of well-being in undergraduate students in Poland through the validation of the Bergen Facebook Addiction Scale (BFAS). Results showed a relation between Facebook addiction and impoverished well-being in terms of impaired general health, decreased sleep quality, or higher perceived stress (Atroszko et al., 2018). Calvo and Carbonell (2018) designed an experimental and longitudinal study aimed at increasing the well-being of individuals experiencing homelessness. Their starting point was the use of information and communication technologies, more specifically, the use of social networking sites by homeless people, and they used Facebook and four scales measured at different points. The findings of this study indicate that Facebook as an essential element that can improve the psychological well-being and socialization of the homeless (Calvo and Carbonell, 2018).

Hussain et al. (2019) used four questionnaires and an eye-tracking methodology to study social networking site (Facebook) use and its relation to different indicators of well-being (mental well-being, depression, anxiety, stress, and self-esteem). The study of the interactions with the areas of interest of the interface of this social network found that Facebook addiction, personality variables, and the Facebook features that individuals interact with are determinant in the individual outcomes related to well-being variables. Kim and Stavrositu (2018) used one online questionnaire in the United States and South Korea to study the role of culture in the relationship between feelings on Facebook and their correlates with psychological well-being. Their results suggest that “experiencing culturally fit emotions stemming from social interactions on Facebook appear to make users fulfill central cultural mandates” (Kim and Stavrositu, 2018, p. 86). Park and Min Baek (2018) used one national survey dataset to study how social comparison (SC)-based emotions shared in Facebook affect individuals’ psychological well-being. They found that “psychological well-being was indirectly influenced by users’ ability-based or opinion-based SC orientations via four types of SC-based emotions [optimism, inspiration, depression, and envy]” (Park and Min Baek, 2018, p. 90).

Marino et al. (2018) used a systematic review of the literature and meta-analysis to study associations among problematic Facebook use, psychological distress, and well-being among teenagers and young adults. The authors could not establish directionality between Facebook use and psychological distress and well-being due to the cross-sectional design of the studies in their sample. Plunz et al. (2019) used a method that allows the capture of geolocated tweets and identifying users who tweeted in parks. The objective was to compare whether tweets made by the same group of Twitter users when inside parks showed more positive sentiments than when they were outside parks (Plunz et al., 2019). The study found that “in-park tweets express less positive sentiment as compared to tweets outside of parks, but park visitors in the other boroughs of New York City [other than Manhattan] generate more positive in-park tweets as compared to those outside of parks” (Plunz et al., 2019, p. 235).

Chen and Ren Huang (2019) analyzed the relationship between religion and the happiness indicator of well-being in Christianity and Buddhism through Twitter by analyzing the proportion of words related to social, cognitive, and affective processes. These authors found psychological differences between Christians and Buddhists through the word analysis, with Christians found to be more social and positive, while Buddhists were more cognitive and negative. Morry et al. (2018) used Facebook to design an experiment regarding the way people construe relationship judgments when exposed to a friend’s or colleague’s Facebook profile in contrast with their relationship and well-being. The authors started from the relationship between social comparisons and personal well-being theories. They found that “individuals may react more strongly to comparisons with close others as opposed to distant others” (Morry et al., 2018, p. 140), which places the focus on the role of Facebook as a key factor. On a different note, but still concerning quantitative methodology, Penchalaiah et al. (2019) proposed applying probability theory to tweets generated by users for the early detection of suicide intentions. Authors used experiments for early detection of suicide warning signs through Twitter’s streaming API, and they managed to “capture warning signs in text compared to traditional machine learning classifiers” (Penchalaiah et al., 2019, p. 1).

We also found a qualitative study that analyzed tweets related to leading brands of wearables in the US to explore perceptions and reactions toward wearable devices that improve health and well-being (El-Gayar et al., 2019). More specifically, the authors used supervised learning, sentiment analysis, and automated content analysis, which demonstrated “the relevance of persuasive design features such as dialogue, credibility, and social support, through to various degrees,” among other uses (El-Gayar et al., 2019, p. 3858).

The evidence in these articles presents the impact of the use of technology in people’s lives. This study aims to offer a methodology that contributes to measuring the social impact of well-being research in people’s lives. For this purpose, we build on a previous study in the area of the social impact of research in social media (Pulido et al., 2018). More specifically, we placed our attention on two social media platforms that attract numerous studies and users worldwide (Facebook and Twitter) to extract those pieces of evidence of the social impact of the research on well-being shared in social media using the SISM methodology.

### Conceptual Framework of the Social Impact in Well-Being Research Applied

This study is inspired by the perspective of the social impact of research (Reale et al., 2018). In doing so, this article is a pioneer in the study of the social impact of well-being research on individuals, and it is the first to apply a specific methodology to measure this social impact of well-being research in psychology. According to Reale et al. (2018), social impact of research takes place “whether researchers generate interventions based on research findings and provide evidence on resulting social improvements, or whether researchers identify actions that have a positive impact on society and analyse their features to create possibilities for transferability” (305). Moreover, to achieve social impact, researchers must create spaces for dialogue with and for the participation of other researchers, stakeholders, and the public, as this is the best way to ensure that they respond to societal needs in their research (Reale et al., 2018). This concept of social impact applied to social media research led to the development of the SISM methodology (Pulido et al., 2018), and this SISM methodology guides the present study on the social impact of psychological research on well-being in social media.

The social impact of research is fundamental and desirable in all scientific disciplines. In psychology, where research is directly conducted on individuals, social impact is even more important because of the immediate effect that it has of either improving the lives of many people or, on the contrary, harming them. For this reason, we chose to study how the SISM methodology applied in psychological research enhances the visibility of the evidence of the social impact of research on well-being.

Thus far, we have seen that the emerging literature on social media has an enormous potential to change people’s lives for better or for worse, and that is why social media research is needed even more. From the researchers’ perspective, social media offers new possibilities for the dissemination of scientific research, and for its meaning dissemination through social networks, which is also known as altmetrics (Ortega, 2018). The fact that the public has access to this information that is ready to use increases the chances of achieving a social impact from research. This possibility, coupled with big data analysis, data mining, and other related techniques, offers the possibility of measuring and evaluating the scientific and social impact of research. Therefore, altmetrics have opened an avenue for measuring the impact of research on society, and in the future, altmetrics could help us study research interactions and communications (Erdt et al., 2016). From the perspective of individuals, social media is a source of collective opinion from which they can learn and make informed decisions in all areas, especially with regard to health and well-being; thus, the information provided in social media can greatly affect an individual’s health (Li and Sakamoto, 2014). According to Li and Sakamoto (2014), collective opinion may act as a filter for deterring the spread of false information and may create a new socially accepted norm that individuals follow even if it contradicts their strong belief.

Making visible the social impact of the research on well-being through the analysis of the social media data available is a step forward in how to measure this impact. Carey et al. (2019) call for the use of metamethods in psychology that can focus on the strengths and weaknesses of different programs or interventions to design better programs and interventions that serve individuals’ needs. The Thrive at Work well-being program has designed a trial to assess the relationship among fiscal incentives, awareness, and the increase in health and well-being offerings at SMEs in the United Kingdom (Thrive at Work Wellbeing Programme Collaboration, 2019). McGhee et al. (2015) propose the use of a longitudinal survey of children in state care to better understand children’s pathways in the system and improve the response of public policy, evaluation, and research on the multi-professional interventions required by this vulnerable group. Espinosa-Montero et al. (2016) developed an instrument to measure the relationship between water consumption and well-being using empirical data from a low-income adult population in an urban area, which can be useful in other contexts. Finally, Robinson et al. (2019) found a positive impact of engaging with books on the well-being of children and young adults with severe and profound learning disabilities, which is in line with what the Children and Families Act of 2014 required in England. What all these examples have in common is the fact that they are oriented to responding to societal needs, sometimes by including stakeholders’ knowledge about their daily life experience. The contribution of our article is to demonstrate that the SISM methodology provides an additional method to make visible the social impact of well-being research.

Considering these previous contributions and the goal of this study, the research questions we raise are: is there any evidence of social impact from well-being research shared on social media? If so, are these examples of potential or real social impact? What are the main contributions identified?

## Materials and Methods

Social Impact in Social Media is a novel methodology in social media analytics for the evaluation of the social impact of research (Pulido et al., 2018) developed under IMPACT-EV, a research project funded under the Framework Program FP7 of the Directorate-General for Research and Innovation of the European Commission. One of the contributions of this methodology is to distinguish those messages published in social media profiles that are classified as dissemination messages from those messages that constitute evidence of the real or potential social impact of research. This contribution is significant, because we are moving toward the evaluation of research efforts that are measured by the social impact achieved, which ultimately refers to how well we are contributing to improving people’s living conditions. This methodology could be applied in any scientific area. In the present case, we have applied it to psychology, specifically in the field of well-being research.

### Data Collection

The data collection, analysis, and dataset created applies the ethical recommendations concerning social media data research of the European Commission (2018). The first step in the data collection was to select the research projects aimed at studying well-being or related areas to be analyzed. In the selection of these research projects, we applied the following criteria:

Criterion 1. Selection of 10 competitive research projects on well-being that publicly display their data on social media. We selected eight research projects funded by the FP7 (7th Framework Programme) and H2020 of the European Commission. Two research projects were selected from two universities from the United States due to their contribution to well-being research. Most of the H2020 projects selected are ongoing projects, but they have already presented research findings in the field of well-being research that are relevant for this study.

Criterion 2. The period of the selection of the social media data is the period from when the first message was published, and the data was available in their profile until August 2019.

Criterion 3. The social media data collected are from Twitter and Facebook. In this study, we applied the criterion that the research project must be present in one of these two social media platforms. In the case of Facebook, we selected Facebook pages with public posts.

The second step was to define strategies for capturing the tweets and Facebook posts of the research project. In this research, we applied two different strategies. The first one was to collect, through NVivo software, the corresponding social media data of the official research project accounts on Twitter and Facebook. The second strategy was to collect those tweets that mention the keyword that defines the research project selected. It is necessary to combine these two strategies to collect the maximum of social media data published in relation to the research project; to choose only one limits the results. Table 1 summarizes the number of tweets and Facebook posts that we collected from each research project using the different social media platforms and strategies previously defined.

**TABLE 1:** Number of tweets and Facebook posts collected for each search strategy.

		Strategy 1		Strategy 2
Project	Program/zone	N. Tweets –	N. Facebook	N. Tweets/
		profile account	page – project	keyword
P1	H2020/Europe	–	–	91
P2	FP7/Europe	58	–	36
P3	United States	202	19	141
P4	H2020/Europe	51	–	–
P5	H2020/Europe	–	105	14
P6	H2020/Europe	90	–	–
P7	United States	–	33	392
P8	H2020/Europe	55	–	29
P9	H2020/Europe	220	–	–
P10	H2020/Europe	23	–	–
	Total	699	157	703

The total amount of the data collected through strategy 1 and strategy 2 is 1,559 messages (1,402 tweets and 157 Facebook posts).

### Data Analysis

We analyzed all the tweets and Facebook posts collected (1402 tweets and 157 Facebook posts) to first calculate the ratio of evidence of social impact to the total amount of data collected. To do so, the first step was to apply a content analysis of the tweets and Facebook posts selected, and the second step was to calculate the corresponding ratio, which is called the SICOR – social impact coverage ratio (Pulido et al., 2018). The result is expressed as a percentage. In this paper, we use the following SICOR for Twitter and Facebook.

$$S\,I\,C\,O\,R=\frac{\sum_{i=1}^{n}\gamma_{i}}{\sum_{i=1}^{n}T_{i}}=\frac{\gamma_{1}+\gamma_{2}+\cdots +\gamma_{n}}{T_{1}+T_{2}+\cdots +T_{n}}$$

where:

• γi is the total number of messages obtained about project *i* with evidence of social impact on social media (in this case, Twitter and Facebook);
• Ti is the total number of messages from project *i* in social media (in this case Twitter, Facebook);
• n is the number of projects selected.

The third step carried out in this analysis was to identify whether the social impact evidence selected was of real or potential impact and whether there was quantitative or qualitative evidence. The main contributions were identified under the criterion of having captured a lot of interest from citizens on social media. This interest is measured by considering the following criteria: the research captured the attention of people who are not directly involved in the research, and these people have published the social impact evidence or have retweeted, liked, shared, or commented on it.

### Analytical Categories and Codebook

The research team that conducted this analysis has expertise in the evaluation of the social impact of research in social media. The analytical categories were previously defined and tested in the current sample. The unit of analysis is the full complement of information available in the message analyzed (tweet or Facebook post). Therefore, the links provided were also examined. Sometimes the evidence of social impact is provided in the message, but at other times, the message that is posted is an introduction, and the evidence is presented in an attached link. The analysis of the user profiles has some limitations. The information in the bio is the unit of analysis of the profile, but some of the bios are empty, and access to others is restricted for privacy reasons. In this sense, we considered in our study only those profiles that are entirely public to measure citizen interest, and for privacy and ethical reasons, we cannot show the profiles. The codebook used in the analysis can be seen in Table 2.

**TABLE 2:** Codebook SISM used in this research.

Code	Description
DI	Tweet or Facebook post of which the main goal is to disseminate the research.
OT	Tweet or Facebook post that contains types of messages other than dissemination and not evidence of social impact, sometimes congratulations, jobs offers, personal interactions, etc.
ESISM	Evidence of social impact is a research result that contributes to the achievement of an objective of society defined by the corresponding institution, for instance, the UN sustainable development goal 3 – Good health and Wellbeing. Evidence can be of potential impact or social impact already achieved.
QUANESISM	Quantitative evidence of social impact. The evidence provided gives quantitative information about improvements obtained through the implementation of the research results. Evidence can be of potential impact or social impact already achieved.
QUALESISM	Qualitative evidence of social impact. The evidence provided gives quantitative information about improvements obtained through the implementation of the research results. Evidence can be of potential impact or social impact already achieved.
POTENTIAL SI	Potential social impact means that research findings have a potential social impact because of the type of evidence the research team aims at identifying and links with societal goals.
REAL SI	Real social impact means that there is already evidence for the social impact of the research findings.

### Inter-Rater Reliability (Kappa)

The analysis of the sample selected was conducted following a content analysis method in which reliability was based on a peer-review process. Each tweet and Facebook post were analyzed to identify whether it contained evidence of social impact (ESISM) or was another type of communication (DI – Dissemination, OT – Other) according to the codebook defined previously. A second level of the analysis was to perform a qualitative analysis of those messages coded as ESISM to detect qualitative and quantitative evidence and identify whether they present real or potential social impact. Once this step was completed, the researchers selected the main pieces of evidence considering the interactions received (likes, shares, comments, retweets), and after that, they analyzed those public user profiles that were involved in the interactions. The first step was to deliver the codebook *a priori* to the researchers in charge of the analysis. To calculate the reliability of the analysis, we used inter-rater reliability in examining the level of agreement between the two raters with regard to the assignment of the categories defined through Cohen’s kappa. We used Cohen’s kappa calculator to calculate this coefficient. The research team analyzed the coding agreement and further classified the sample with regard to dissemination (DI = 1), evidence of social impact (ESISM = 2), or other type of message (OT = 3). There were 16 messages coded with different values, and they were excluded from the final sample analyzed. The result obtained is 0.89%. By interpreting this number according to the Cohen’s kappa coefficient (see Supplementary Table S2), our level of agreement is reliable. Once this review was complete, the final sample was composed of 1543 tweets and Facebook posts.

In this paper, we show the results of the sample classification in the three categories. Also, we provide the results of a second analysis focused on the potential and real social impact, with an example of qualitative and quantitative evidence in the last section that illustrates the selected contributions.

## Results

To answer the research questions defined previously, in this section, we divide the results into three parts. The first focuses on the question of whether there is any evidence of social impact from well-being research being shared in social media. The second focuses on the question of whether the evidence shared contains information about real or potential social impact, and the third focuses on the main contributions identified based on the results regarding the detection of real social impact.

### Evidence of the Social Impact of Well-Being Research Shared in Social Media

With the analysis complete, we can confirm that there is evidence of the social impact of well-being research shared in social media (15.7%). We found a higher percentage of the presence of ESISM (evidence of social impact) in this study than in a previous study, where the highest percentage of ESISM for a project was 4.98% (Pulido et al., 2018). In the current study, the project with the highest percentage had 27.5%.

Next, the results are explained in global and detailed ways. Table 3 illustrates the coverage percentage of messages coded based on the codebook (DI, ESISM, OT).

**TABLE 3:** Percentage of types of coded message.

Type of message	Number of references	Percentage
DI	880	56.9
ESISM	244	15.7
OT	429	27.5

The type of message (tweet or Facebook post) with the highest percentage is dissemination (56.9%), while the percentage of ESISM (15.7%) is lower. This result is in line with previous research results that indicated that tweets and posts are mostly linked to dissemination goals.

Table 4 illustrates the result of the SICOR considering the results of the sum of the ESISM based on the 10 research projects in relation to the total number of tweets and Facebook posts collected, classified by the type of strategy and type of social media platform.

**TABLE 4:** Global SICOR results.

Type of strategy	SICOR – Social Impact Coverage Ratio
Strategy 1 – Profile	13.8%
Strategy 2 – Keyword	17.9%
**Type of social media platform**	
Twitter	15%
Facebook	21.7%

The results extracted by the strategy indicate that the SICOR is slightly higher in strategy 2 than in strategy 1. With regard to the type of social media platform, although we collected more tweets than Facebook posts, the posts have higher SICOR than the tweets. This result indicates that the number of tweets or Facebook posts that are collected is not relevant to finding evidence of social impact, which is in line with the previous research conducted under the SISM methodology.

Table 5 illustrates in detail the SICOR results extracted for each research project selected. As shown in the table, there are three projects with more than 20% evidence of social impact (P4, P5, and P7). Two of these projects are recent research projects funded by the H2020 Framework Programme, while P7, which has the second-highest percentage, belongs to one of the United States research projects that is one of the most relevant studies in well-being research. Regarding the other projects, we found that three of them have more than 10% evidence of social impact (P2, P3, and P6), three of them more than 5% (P1, P8, and P10), and one has less than 5% (P9).

**TABLE 5:** SICOR results for each project.

Project	Program/zone	SICOR
P1	H2020/Europe	9.9%
P2	FP7/Europe	14.9%
P3	United States	10.8%
P4	H2020/Europe	27.5%
P5	H2020/Europe	23.5%
P6	H2020/Europe	12.2%
P7	United States	26.4%
P8	H2020/Europe	8.3%
P9	H2020/Europe	3.6%
P10	H2020/Europe	8.7%

### More Real Social Impact Than Potential Social Impact Found in Selected Projects

We have identified 244 tweets and Facebook posts that contain evidence of social impact. When we analyzed the content of these messages in depth, the result was that there were more detailing real social impact (83%) than potential social impact (17%). This result confirms the idea that applying SISM (see Supplementary Table S1) to identify evidence of social impact is a quick way to capture relevant pieces of evidence of social impact that researchers and citizens are sharing in the online public space. Table 6 shows the number of tweets and Facebook posts collected and the corresponding percentages in relation to the total number of ESISMs collected.

**TABLE 6:** Percentage of real and potential social impact identified in the ESISM sample.

Code	Number of Tweets and Fb/posts found	Percentage
ESISM	244	100
POTENTIAL SI	42	17
REAL SI	202	83

We elaborated Table 7 to display which projects have more real social impact than potential social impact and to specify the results classified in both categories. In this way, we can detect which project results it would be interesting to analyze in depth to demonstrate the evidence of social impact. The percentage indicated corresponds to the total amount of social media data collected for the project.

**TABLE 7:** Results of real and potential social impact for each project in relation to the total amount of the ESISM sample.

Project	Code	N. Tweets and Fb/posts	Percentage
P1	POTENTIAL SI	7	2.9
	REAL SI	2	0.8
P2	POTENTIAL SI	4	1.6
	REAL SI	10	4.1
P3	POTENTIAL SI	11	4.5
	REAL SI	28	11.5
P4	POTENTIAL SI	5	2.0
	REAL SI	9	3.7
P5	POTENTIAL SI	6	2.5
	REAL SI	22	9.0
P6	POTENTIAL SI	0	0.0
	REAL SI	11	4.5
P7	POTENTIAL SI	0	0
	REAL SI	112	45.9
P8	POTENTIAL SI	4	1.6
	REAL SI	3	1.2
P9	POTENTIAL SI	4	1.6
	REAL SI	4	1.6
P10	POTENTIAL SI	1	0.4
	REAL SI	1	0.4

Considering these results, we can detect which of the projects has the greatest percentage of real social impact (P7), with 45.9%. The second-highest (P3) is considerably lower than the first, with a result of 11.5%, while the third one (P5) is close to the second, with 9%. The fourth (P6) is half of the third, with 4.5%, and the last one (P2) is close to the fourth, with 4.1%. In the next section, we analyze the evidence of the social impact of the five projects with the highest percentage of real social impact.

### Main Contributions Identified

Before beginning to explain the different examples collected from these projects, we would like to mention that the evidence of social impact that we will deliver could be in the same tweet or Fb/post or in a link delivered with that tweet or Fb/post, as we explained in the prior study (Pulido et al., 2018). Considering the results explained in the previous section, we selected the following examples:

#### The Quality of Relationships Directly Influences Health

The first project (P7) belongs to the longest study of adult development, led by researchers of Harvard University. This study has been in place for more than 80 years, and there are results available from the first part of the study focused on people who began the study in the 1940s. Now, the researchers are leading a second part of the study that is focused on the second generation, the children of the participants of the first cohort. Evidence of the social impact of this study is the most tweeted and shared by citizens of all the samples collected in this study. As we have shown in Table 7, this project has the highest percentage of real social impact because there are more tweets shared directly by citizens than in the other projects. Citizens that tweet this evidence have been impressed to find out that health depends more on strong and meaningful relationships than on fame, money, or social class. There is no doubt that this evidence has captured much citizen attention. Here are some examples of the messages related to this project.

The first two examples epitomize this qualitative evidence:

“Harvard Study of Adult Development has tracked the lives of 724 men for 79 years and found that flourishing in life is a function of close ties with family, friends, and community. It had nothing to do with fame, wealth, social class, IQ, genes, etc.” (REAL SI, P7, REF 87).

“’Close relationships, more than money or fame, are what keep people happy throughout their lives. and are better predictors of long and happy lives than social class, IQ, or even genes,’ concludes the 80 Year Harvard Study of Adult Development.” (REAL SI, P7, REF 3).

The following quote is also an example of how being aware of this qualitative evidence of social impact can encourage one to apply the study results in one’s own life:

“Watched a video today that said a Harvard study on Adult Development conducted over 75 years shows that what keeps people living longer and happier is the quality of their social relationships. Put the electronic gadgets down and actually engage in meaningful interactions. #HealthyLiving.” (REAL SI, P7, REF 20).

Another finding highlighted how loneliness negatively affects health and the relevance of applying the evidence of improving health through strong relationships:

“Loneliness kills. The big finding from the Harvard Study of Adult Development. If you want to live a happy and healthy life, you have to prioritize having strong social connections and relationships.” (REAL SI, P7, REF 9).

Finally, this example highlights one of the pieces of evidence regarding the quality of connections. Similarly, the example encourages the application of this evidence in daily life:

“It is never a question of IF there are connections in life, it is only a question of the quality of those connections. This study shows us that the better that quality, the better our life and our health long term. What more do we need to know?!? Check out my podcast for more about happiness too!” (REAL SI, P7, REF 7).

#### Early Detection of Depression Symptoms Through the Analysis of Language in Social Media Improves Prevention

P3 belongs to the World Well-Being Project (WWBP), led by researchers from the University of Pennsylvania. These researchers are leading a pioneering research study of scientific techniques for measuring psychological well-being and physical health based on the analysis of language in social media. The focus of their research is on those psychosocial processes that affect health and happiness and how to improve them. In this case, the examples provided links to explore the evidence identified. Some of the examples selected present evidence of how the analysis of social media predicts earlier symptoms of depression that could be used to help prevent depression before these symptoms worsen:

“Researchers from the WWBP analyzed social media with an AI algorithm to pick out linguistic cues that might predict depression. This may lead to early detection and treatment for many. bit.ly/2E205OL.” (REAL SI, P3, REF 1).

“We show that Facebook statuses can be used as a (rough) screening technology for depression as recorded in medical records – AUC.69 – article is open access. #depression #BigData @WWBProject https://t.co/sLQpCTfPaC.” (REAL SI, P3, REF 7).

#### Engaging With Meaningful Social and Intellectual Interactions Impacts Brain Health

The third research project is LIFEBRAIN, funded by the H2020 program of the European Commission. The aim of this project, according to its webpage, is “to identify determinants of brain, cognitive and mental health at different stages of life and establish a solid foundation of knowledge for understanding how brain, cognitive and mental health can be optimized through the lifespan.” This research is an ongoing project that will end in 2021, but the researchers have already identified evidence of social impact. Some of this evidence comes from researchers who belong to their own network, and the rest comes from other colleagues who are contributing to this research field. The projects’ research team uses a newsletter to share the evidence, quoting the studies referenced and using good science communication language to reach more people. These posts have more interaction than in the other cases. The examples selected are from the project’s Facebook page. Some of the Fb/posts contain this evidence; for instance, the relevance of being involved in social, physical and intellectual activities in middle age has a direct impact on brain health in old age:

“Lifebrain researcher Professor Rik Henson at the University of Cambridge presented the recent results obtained within the CamCan study cohort to the Lifebrain research group at the University of Barcelona this week. One interesting finding is that middle-aged people who participated in higher levels of social, physical and intellectual activities had better thinking ability in old age than those who undertook fewer of these midlife activities, despite age-related reduction in their brain sizes. Read more about the results of the Cambridge cohorts here: https://www.lifebrain.uio.no/publications/e-newsletters/midlife.html.”

The quantitative evidence could be explored inside the link attached, which includes a reference to the research publication where the results are available.

Meaningful leisure-time interactions also have an impact on brain health:

“Leisure activities count for your brain health! Frequently engaging in social and intellectually stimulating activities, such as meeting friends or family, playing board games, and reading, are linked to better brain health in old age. This is a finding from a current review performed by Lifebrain researchers at the Department of Psychiatry, University of Oxford. Read more about the impact of leisure activities on brain health in the latest Lifebrain e-newsletter: https://www.lifebrain.uio.no/publications/e-newsletters/leisure.html.”

For women, there is an interesting piece of evidence reported by this project. Researchers found that women have healthier brains in gender-equal countries; thus, equality affects their well-being as well:

“Women have healthier brains in gender equal countries. In countries that promote women’s equality and participation in society, women have a better chance of keeping their brains healthy in later life, according to new research from the Norwegian Institute of Public Health. Read our monthly e-newsletter here: http://mailchi.mp/95211d178033/lifebrain-horizon2020-project-e-newsletter.”

#### Positive Mental Health Self-Ratings Improve Future Mental Health

The fourth project is CAPICE, funded under the H2020 project by the European Commission. This is an ongoing project that will also end in 2021, but the researchers have already shared evidence of social impact on the well-being and mental health of children and adolescents in Europe. One of the pieces of evidence shared relates to an advance in improving the treatment and prevention of mental health: that the positive self-rating of one’s own mental health has a positive impact on future mental health:

“Self-rating mental health as ‘good’ predicts positive future mental health https://t.co/G4YzTkNnfl #mental-illness #depression #psychologicaldistress #InvestEUresearch @GamianE @capice_project @EU_H2O20, https://t.co/vKqdElsgrA.”

If we follow the link, we find more information about the findings of this study published in the Journal of Health and Social Behavior. Researchers found that for 62% of people with a mental health problem, if they rated themselves in a positive way, they faced their health issues better. Besides, the researchers found that individuals who evaluated their mental health as good had 30% lower probabilities of having a mental health problem, being an example of quantitative evidence. According to the researchers (McAlpine et al., 2018), “even without treatment, persons with a mental health problem did better if they perceived their mental health positively” (1). In fact, researchers involved in this study show that self-rated mental health had an independent positive influence on future mental health and highlight other elements that agree with previous evidence quoted in this section, for instance, the benefits of meaningful relationships and maintaining a sense of purpose and belonging in life.

#### The Use of Machine Learning Algorithms Improves the Accuracy of Mental Health Predictions

The last project selected is PRONIA, funded by the FP7 project of the European Commission, which focused on research on personalized prognostic tools for early psychosis management. One of the aspects of well-being research is to prevent mental illness and promote people’s well-being. In this sense, this research team shows how well-trained machine learning algorithms better predict mental health outcomes because the data offered are based on evidence. The following example of quantitative evidence reveals this type of accuracy:

“PRONIA Project @PRONIA_EU ⋅ 14 Nov 2018 #PRONIA managed to show in a #MultinationalStudy that #MachineLearning algorithms perform better than doctors at predicting #MentalHealth outcomes, with up to 82% accuracy. Listen to the radio interview with @koutsouleris & @StephenWood8 on @abcnews! ∼ https://www.abc.net.au/radio/programs.”

This last example shows how effective early treatment of youths can prevent mental health problems later in life:

“Exactly!! Choosing where & when to invest precious dollars in #mentalhealth care is not a zero-sum game. Effective treatment for #youth can bend the life curve positively AND prevent #disability when older. It pays off in multiple ways! #onhealth #cdnhealth https://t.co/Gxdq22BGzA.”

## Discussion

The European research projects selected are mostly ongoing projects, but since their very beginning, they have been sharing evidence of social impact in their social media channels. This result could be due to the current evaluation system of the European Framework Programme (van den Besselaar et al., 2018), where social impact is one of the crucial criteria for being funded and evaluated. The other two research projects selected that contain a lot of evidence of social impact are research projects with a long trajectory of conducting research in the same field, and this is one of the reasons that they have more evidence. However, one of the common criteria is that all the projects selected have research goals related to societal goals. Therefore, all the projects address, for instance, priorities defined by the SDG3 Health and Wellbeing for All (UNDP, 2017), one of the sustainable development goals defined by the United Nations. The topics covered are in line with the most common issues investigated by the academic community, according to Kapoor et al. (2018). The difference with this previous study is that we have focused on that evidence that contributes to improving people’s lives. Thus, merely descriptive research results are out of the scope of this paper.

Another detail observed in this study is critical for the improvement of the living conditions of citizens. We observed in some tweets and Fb/posts by citizens that they highlight the evidence of social impact that guarantees an improvement in their life and encourage others at the same time to implement changes supported by the evidence in their daily lives. This finding could be related to the evidence founded by Park and Min Baek (2018), where users’ abilities and emotions indirectly influenced psychological well-being. But this finding also needs other contributions to understand why users encourage others when they find evidence for improving their lives. For instance, Hooker et al. (2019) provide evidence of the direct impact of being engaged in meaningful activities on psychological well-being. One possible explanation is that social media users that have interacted with evidence of social impact created a meaningful understanding that needs to be shared with others.

This is an important finding, since fake news affects the health of many people, according to Merchant and Asch (2018). Spreading evidence of how to improve well-being, which directly affects health, is crucial for guaranteeing the achievement of SDG3: Health and Well-being for All. This approach is particularly significant with regard to the evidence found in P7. When citizens get to know that science contributes to improving their lives, they tweet, post, like, share, and comment. This result is an indicator that researchers have connected with citizens due to the relevance of their findings, which guarantees an improvement in people’s lives.

The research projects analyzed show the relevance of well-being research in our times. Nonetheless, the projects face one large obstacle. This obstacle is the fact that in this field, there are many non-evidence-based assertions. One way to deal with them is to share evidence of social impact in social media to reach out to more people who are not normally familiar with the academic environment.

The limitations of this study are mainly due to the data restrictions, which were updated by the General Data Protection Regulation (EU) 2016/679 (GDPR). Twitter and Facebook have correctly updated their data protection under this law. Programs such as the one used by NVivo respect these updates. The information extracted respects this legal framework because it derives from the public data that users have consented to share, and any private data remains private according to the ethical guidelines in social media data research. Therefore, our dataset collects permissible public information, and anonymity is guaranteed. It is important to note that some tweets and Fb/posts are not collected because they are not provided by the platforms due to privacy reasons. In this sense, we are aware that there are more tweets and Fb/posts, although the dataset collected already displays evidence that social impact is shared in social media despite these limitations. Another limitation included in the study is that the data collected should be contrasted to avoid the possibility of manipulation or misuse. For this reason, all evidence of social impact found is checked for a reference of scientific evidence that guarantees that is not false information.

Finally, we would like to highlight one finding that was not mentioned before. Some of the social media channels of the research projects not only shared their own evidence of social impact but also shared evidence from the research of other colleagues who are also providing key contributions in the same field. This detail is of note because when scientists prioritize bringing together findings that can improve the well-being of the people, science can advance faster and better.

## Conclusion

Considering the research questions elaborated in this study, we can conclude with the following results. The application of SISM in the analysis of 10 competitive research projects on well-being research confirms that this type of research produces evidence of social impact that is shared in social media. We have obtained more real social impact evidence than potential evidence; this fact implies that the research selected is already contributing to improving people’s lives. The main contributions identified are those on how the quality of relationships has a direct impact on the quality of health over a lifetime and that engagement in meaningful social and intellectual interactions supports brain health. Moreover, the positive self-rating of one’s own mental health promotes future mental health, so this is an important variable for the treatments in this field. Technology is used to prevent mental health problems, such as through early depression detection and more accurate detection of mental problems. Both improve treatment and prevention, which promotes well-being and better health. These results relate to SDG 3, Health and Wellbeing for All, which is one of the priorities of the Sustainable Development Goals of the United Nations, and have already achieved social impact. Regarding the methodology proposed, we have demonstrated that SISM contributes to focusing on extracting the evidence of the social impact of well-being research and makes more visible how the research results provided improve people’s lives, obtaining social impact. Knowing these results allows advances on how to define future research proposals and on how to collect data on the social impact of current research. Besides, one future line could be to explore how the citizens that know about the evidence of social impact through social media channels applied this evidence in their daily lives and to collect results of this implementation.

## Data Availability Statement

All datasets generated for this study are included in the article/Supplementary Material.

## Author Contributions

TS-M and CP contributed to the conception and design of the study. L-CM selected the literature review. VI and CP selected and analyzed the social media data sample collected and reviewed the data analysis and results sections. L-CM and CP wrote the first draft of the manuscript. TS-M reviewed the final version of the manuscript and the contributions.

## Conflict of Interest

The authors declare that the research was conducted in the absence of any commercial or financial relationships that could be construed as a potential conflict of interest. The reviewer JC declared a shared affiliation, with no collaboration, with one of the authors, VI, to the handling Editor at the time of review.

## Supplementary Material

TABLE S1 SISM-SICOR calculation.

TABLE S2 Kappa coefficient.
